# Structural Analysis of Si(OEt)_4_ Deposits on Au(111)/SiO_2_ Substrates at the Nanometer Scale Using Focused Electron Beam-Induced Deposition

**DOI:** 10.1021/acsomega.3c00793

**Published:** 2023-06-28

**Authors:** Nigel J. Mason, Maria Pintea, István Csarnovics, Tamás Fodor, Zita Szikszai, Zsófia Kertész

**Affiliations:** † School of Physical Sciences, University of Kent, Ingram Building, Room 201, Canterbury CT2 7NZ, United Kingdom; ‡ Department of Experimental Physics, Institute of Physics, Faculty of Science and Technology, University of Debrecen, Bem sq 18a, Debrecen 4032, Hungary; § Laboratory of Materials Science, Institute for Nuclear Research, Bem tér 18/c, Debrecen 4026, Hungary

## Abstract

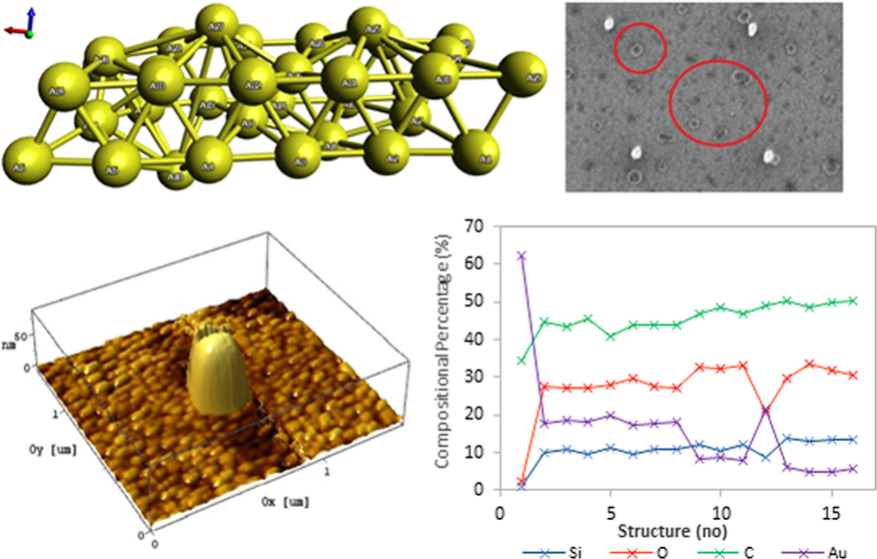

The focused electron beam-induced deposition (FEBID) process was used by employing a GeminiSEM with a beam characteristic of 1 keV and 24 pA to deposit pillars and line-shaped nanostructures with heights between 9 nm and 1 μm and widths from 5 nm to 0.5 μm. All structures have been analyzed to their composition looking at a desired Si/O/C content measuring a 1:2:0 ratio. The C content of the structure was found to be ∼over 60% for older deposits kept in air (∼at room temperature) and less than 50% for later deposits, only 12 h old. Upon depositing Si(OEt)_4_ at high rates and at a deposition temperature of under 0 °C, the obtained Si content of our structures was between 10 and 15 atom % (compositional percentage). The FEBID structures have been deposited on Au(111)/SiO_2_. The Au(111) was chosen as a substrate for the deposition of Si(OEt)_4_ due to its structural and morphological properties. With its surface granulation following a Chevron pattern and surface defects having an increased contribution to the changes in the composition of the final structure content, the Au(111) surface characteristic behavior at the deposition of Si(OEt)_4_ is an increase in the O ratio and a reduction in the nanodeposit heights.

## Introduction

The focused electron beam-induced deposition (FEBID) was first discovered in 1962 when molecular deposits have been found in the mass spectrometer equipment, results of the electron beam-induced chemistry on the molecules of the compound. Since 1962, the technique has increased in importance, in the past 10–15 years progressing to be a viable manufacturing method of circuits, nanomaterials, and semiconductors with implications in a wide range of medical applications. Principles of FEBID techniques have been researched by scientists in Huth et al.,^6^ Toth et al.,^7^ Randolph et al.,^8^ Randolph et al.,^36^ Huth et al.,^37^ Thorman et al.,^38^ and De Teresa et al.,^55^ providing information on nanostructure deposits of Fe(CO)_5_ and Co_2_(CO)_8_ with very high deposition purities of the structures and characteristic values of ∼98 atom % (Fe(CO)_5_)^37^ and 95 atom % (Co_2_(CO)_8_).^55^ High-purity structures of bimetallic compounds with values in the range of ∼80 atom % have been obtained by Ragesh Kumar et al.^56^ at irradiation of deposited layers of HFeCo_3_(CO)_12_ and exposure to room temperature where desorption of the remaining ligands of the CO and H from the resulting HFeCo_3_(CO)_3_ was observed. The FEBID method is mostly used with the SEM deposition of nanostructures and in situ X-ray photoelectron spectroscopy (XPS), atomic force microscopy (AFM), and energy-dispersive X-ray (EDX) studies for DUV/EUVL mask repair or development of complex three-dimensional (3D) nanostructures, the materials ranging from carbonyls, acetates, acetylenes, bromides, chlorides, and iodides to combined bimetallic or trimetallic precursors.

The silica precursors used for substrate deposition (SiO_2_ deposition) range from TEOS to SiCH*_x_*, SiNH*_x_* + O_2_ depending on the deposition process. The most common processes used are CVD and ALD, with a very wide pool of applications in the radiation cancer research and radiation therapy (GSH-responsive mesoporous silica nanoparticles^54^), new material development for catalysis and hydrogenation (Pd-containing hydrogenation nanocrystals immobilized in silica precursors,^50,51^ SiO_2_–CeO_2_ nanoparticles with heat specific tolerance^52^), fiber optics in telecommunications (periodic mesoporous photoluminescent nanocrystal silicon–silica composites^53^), and radiation containment of water, energy generation, and uranium storage (SG-TTA + SiO_2_ with a 98% sorption of uranium(VI)^49^). The beam parameters^58,59^ of the focused electron beam-induced process (FEBIP) in the deposition of SiO_2_ from TEOS were optimized using CASINO simulations and gas-phase studies of the Si(OEt)_4_ fragmentation pathways. Earlier EBID studies^19,23−33,36−38,45,57^ report the deposition of Si(OEt)_4_ at multiple temperatures, as a standalone and mixed with H_2_O,^57^ focusing on the behavior of the structures deposited at different temperatures.

## Experimental Section

### SEM and Measurement Equipment

The SEM C1 with a LEO 1500 series Gemini column scanning electron microscope (SEM) used for deposition of the nanostructures has a performance of 1.0 nm @ 20 kV and WD of 2 mm to 5.0 nm @ 0.2 kV, 2 mm in high vacuum, 1.2 nm @ 20 kV, and 7.0 nm @ 1 kV in variable pressure mode with an acceleration voltage in the range of 0.1–30 kV. The Shottky field emitter electron source is characterized by a high beam brightness and low beam energy spread. The SEM uses an infrared CCD camera with a focusing distance of 1–50 mm and an eight-pole electromagnetic stigmator, a 5-axis eucentric stage with motorized movements on *x* and *y* of 75 mm and on *z* of 55 mm.

The nanostructure analysis was conducted using EDX measurements. The EDX measurements are characterized by an analysis beam voltage of 5 keV. The principle behind the EDX functioning is the process of collision between a molecule or an atom with an electron, with the result of the appearance of a hole in the inner shell, while an electron from the outer shell will take its place emitting a set of characteristic X-rays specific to each element from the periodic table and with a characteristic acceleration voltage, defined by the relation *z*_m_ = 0.033 (*E*_0_^1.7^ – *E*_c_^1.7^)*A*/ρ*Z*. Based on the characteristic X-ray Kα value of the elements in Si(OEt)_4_ (Si, 1.739; O, 0.525; and C, 0.277), a minimum value of 5 kV accelerating voltage of the TEM beam was obtained.

### XPS

X-ray photoelectron spectra were obtained using an Al/Mg twin anode non-monochromated radiation source and a Phoibos100 MCD-5 series hemispherical energy analyzer produced by SPECS (Berlin). The measurements were conducted with Al Kα (*E* = 1486 eV) rays. The sample was examined as received and mounted directly onto the XPS sample holder. The spectra were processed with CasaXPS (http://www.casaxps.com).

### Computational Details

For the electron trajectory simulations, CASINO software version v2.42 and v3.4 were used with focus on beam characteristics of the secondary and backscattered electrons involved in the deposition process of the nanostructures.

### Au(111)/Silica Substrate XPS Characterization

**Figure 1:**
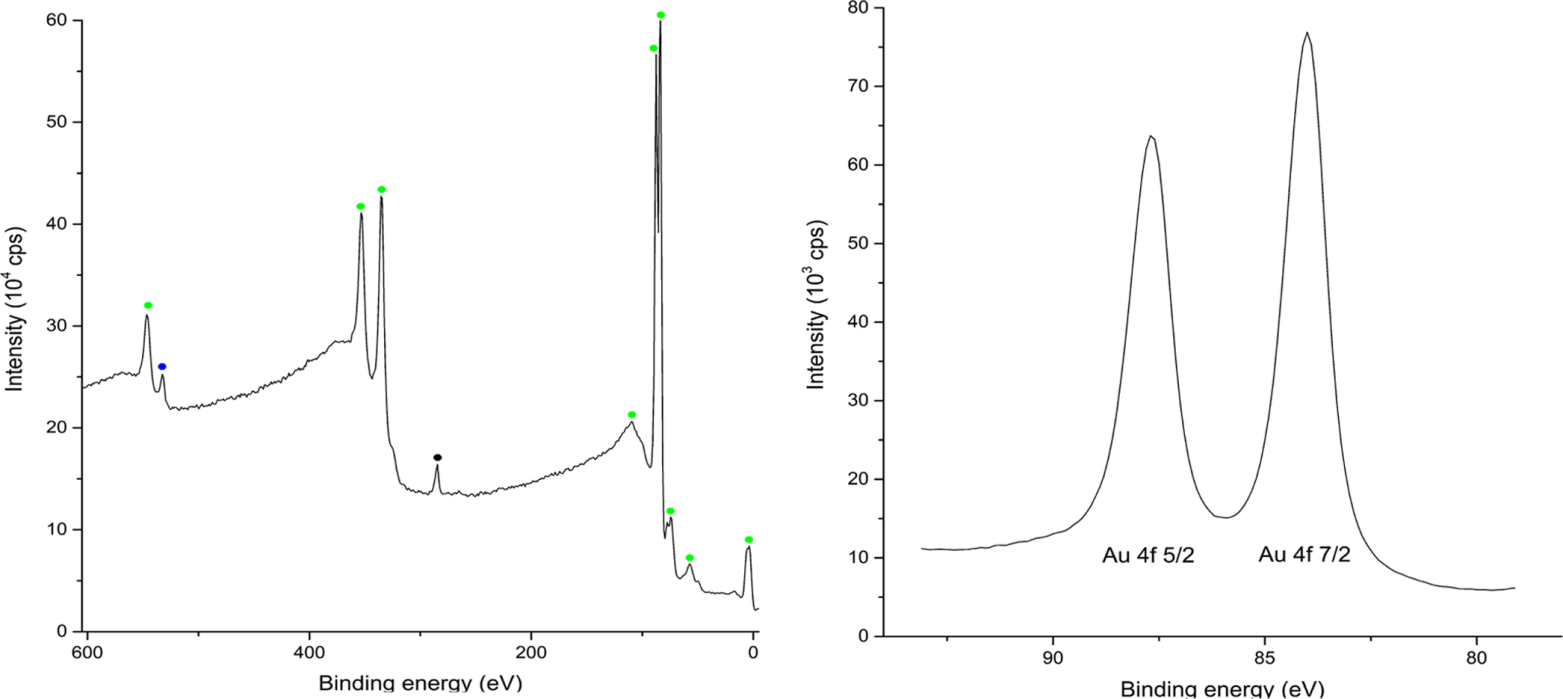
X-ray photoelectron spectrum of the sample and Au substrate. Photoelectron peak assignment: black—carbon, blue—oxygen, and green—gold (left); X-ray photoelectron spectrum of the Au 4f region (right).

X-ray photoelectron spectroscopy was used for post-deposition substrate characterization only, as the instrument setup only allows photoelectron collection from a macroscopic (7 mm × 20 mm) surface area, which is several orders larger than that of the Si-bearing deposits. The survey spectrum shows that the analyzed surface consists of gold and some contaminants (see Figure 1). The elemental composition of the sample surface as determined by XPS has a concentration of 9.46% O_2_, 33.45% C, and 57.09% Au. Adsorbed oxygen and carbon are found on the surface of the sample, as the sample was in prolonged contact with air before the measurement. This technique yields information from the outermost 5–10 nm of the surface. No signals corresponding to silicon were detected via XPS. This was expected, as the peak intensity is a function of surface coverage and the deposits are nanoscale deposits, constituting less than 1 ppm of the total examined surface atoms. The gold substrate itself is composed of the pure element, to the extent that the Au 4f_7/2_ peak appears precisely at the literature binding energy value (84.0 eV) and was used for calibration (see Figure 1).

**Figure 2:**
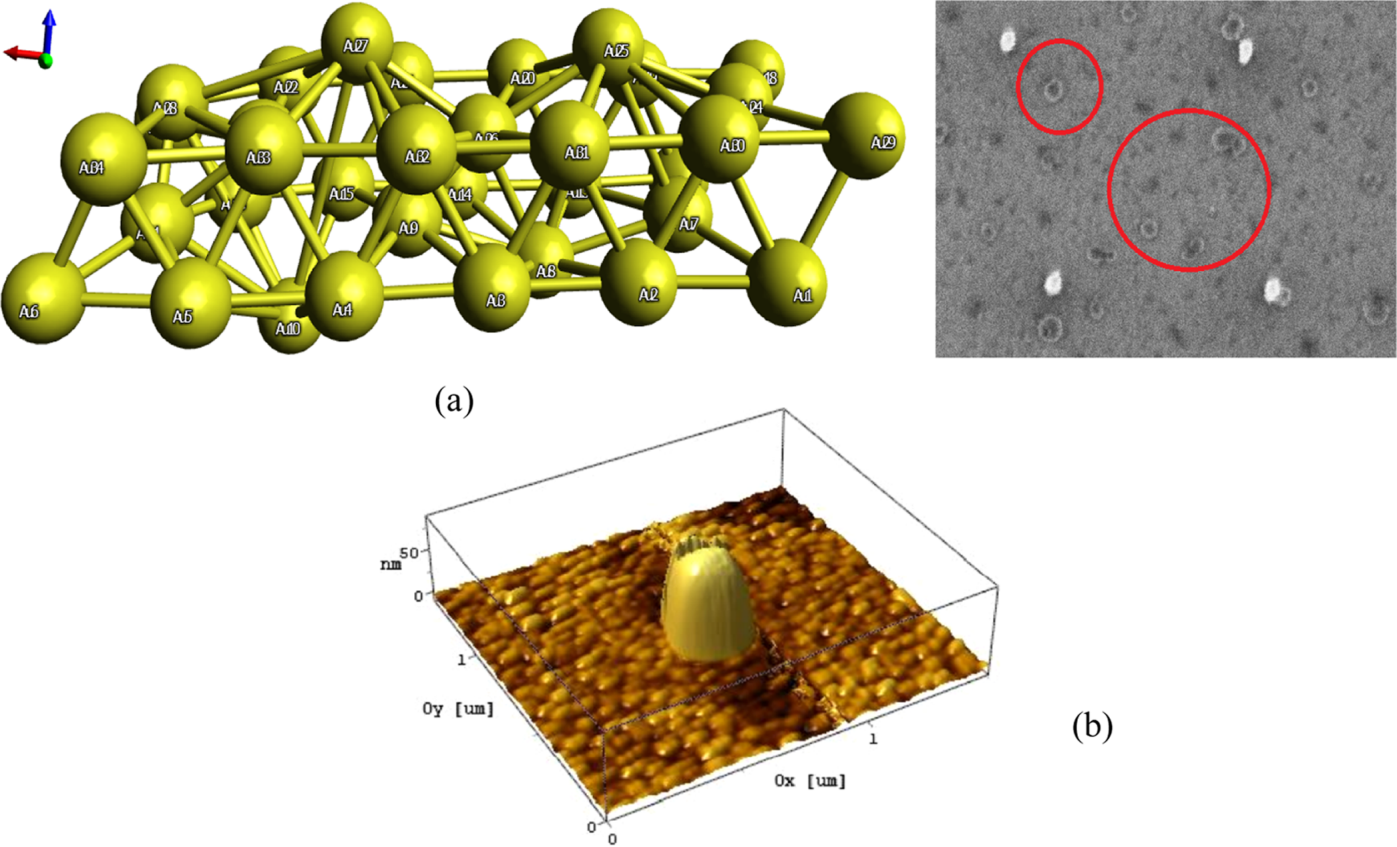
2-layer Au-substrate optimization view with Au27, Au25, Au10, and Au8 sites higher from the axis line (6 × 3 × 2 atoms) (a); 91 nm high pillar shape nanostructure with apparent visible Au-grain surface (b).

The 200 nm (8 months old substrate) and 100 nm (12 h/12 months old substrate) present no signal coming from the SiO_2_ wafer. Simulations of the Au(111) surface^42,43^ reveal the presence of (111), (110), (100), and (211) facets of the substrate. While the Au–Au has a bond value of ∼2.10–3.10 Å and a unit cell length of 4.065 Å × 4.065 Å × 4.065 Å, the underlying SiO_2_ wafer has the Si–O bond distances with a value of 1.58–160 Å and the unit cell of 7.16 Å × 7.16 Å × 7.16 Å, influencing only the organization of the first Au-monolayer on the wafer, forcing the subsequent layers of atoms in a bcc plane configuration. Hanke and Björk^44^ do a reconstruction of the Au(111) substrate for a six-layer slab using a 22 × √3 lattice with a number of 23 atoms fitted in 22 sites for the fcc and hcp configurations with a minimum of six layers of Au atoms needed for a full convergence. The organization of the atoms on the SiO_2_ wafer is following the bcc orientation on the first layer, followed by combined fcc and hcp sites, while the last layers are organized in the fcc configuration. Earlier studies^42,43^ were not able to solve the presence of both hcp and fcc sites as well as the atomistic and electronic degree of freedom important in determining the reactivity of sites and the catalytic activity of the reactions at the surface in the FEBID of the nanostructures. The Au(111) substrates over time present a highly grained surface with visible Au atoms (Figure 2; 8 months old substrate), a reorganization of the surface layers pushing at sites Au atoms with 0.01–0.5 Å higher, as well as the process of integration in the lattice of gas atoms and other atoms from the deposited precursor.

### Si(OEt)_4_ Deposition

The Si(OEt)_4_ was deposited with different dwell times and loops numbers. A set of two deposits were done on the Au(111) substrates. First set was kept 8 months in air after deposition on a Au(111) substrate with visible signs of wear and damage. A second set was deposited on a new Au(111) substrate and analyzed after 12 h from the deposition time and again after 12 months.

**Figure 3:**
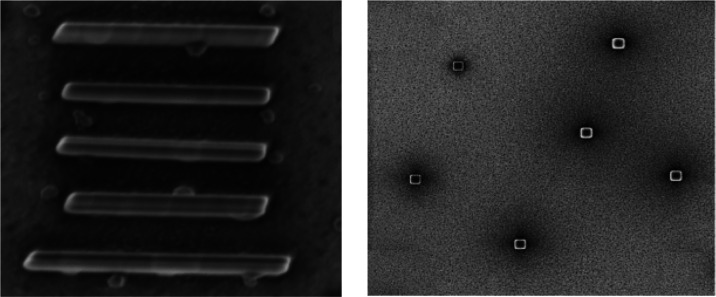
8 months old deposits of Si(OEt)_4_ on the irregular Au surface, 6 lines (0.7 μs; 700 loops) top view (left) and 12 h old deposits of Si(OEt)_4_, 6 points top view (right).

The first set of structures (Figure 3) was deposited using the beam characteristics of 24 pA beam current and 1 keV beam voltage. The parameters of the deposition process are presented in Table 1. The set of measurements contains a number of 2 sets of 6 points and 6 sets of 6 lines, done with variable dwell time constants (presented in Table 1) at 0.7 μs, 0.45 μs, 0.35 μs, and 5 μs respectively. The sample was stored in an aerated container.

**Table 1:** Deposition of Si(OEt)_4_ on Au(111)/Silica, 8 Months Old Deposits

deposit types	Dwell time (μs)	loops (no)	beam current (pA)	beam voltage (eV)
6 points	2.350/12.35/22.35/32.35/42.35/52.35	1k/1.3k/2k/2.5k/3k/1.8k/1.5k/4k	24	1000
6 points	10.325/22.325/24.325/26.325/30.325	1300	24	1000
6 lines	5	1300	25	1000
6 lines	0.7	500	24	1000
6 lines	0.45	500	24	1000
6 lines	0.7	700	24	1000
6 lines	0.35	1300	24	1000
6 lines	0.7	1300	24	1000

The second deposition (Figure 3) was done using the beam characteristics of 28 pA beam current and 1 keV beam voltage. The deposition parameters of the second nanostructures are presented in Table 2. The Au(111)/silica substrate used for the second deposition has different features than the characteristics of the first substrate; it is more homogeneous, and the Au(111) surface deposited on silica has a constant layer thickness of 100 nm. The Au(111)/silica substrate used for the first deposition has a layer thickness of 200 nm and is older than the second substrate, with a higher degree of contamination of the surface coming from dust particles, NO_2_, H_2_, H_2_O, grease, irregularities, and defects. At defect sites, the silica wafer can be seen through the previous depletion of Au(111), and the signals from the SiO_2_ substrate can be obtained. The second deposition contains a number of 2 sets of 7-line deposits at 0.7 and 0.35 μs dwell times and 2 sets with 6- and 7-point deposits with a variable dwell time constant of *t* + 0.2 μs for the set of 6 points and *t* + 1 μs for the set of 7 points. The sample was stored for 12 months in an aerated container.

**Table 2:** Deposition of Si(OEt)_4_ on Au(111)/Silica, 12 h Old Deposits

deposit types	Dwell time (us)	loops (no)	beam current (pA)	beam voltage (eV)
7 lines	0.7	650	28	1000
7 lines	0.35	1300	28	1000
6 points	4.03/4.23/4.43/4.63/4.83/5.03	1000	28	1000
7 points	0.25/1.25/2.25/3.25/4.25/5.25/6.25	2600	28	1000

The deposits were done at a vacuum chamber pressure of 8.2 × 10^–7^ mBar, with a deposition pressure of 1.5 × 10^–6^ mBar and a temperature of the Si(OEt)_4_ precursor introduced to the gas line of −11 °C for the second set of deposits and −25 °C for the first set of deposits.

## Results and Discussion

### Deposit Analysis

Si(OEt)_4_, or under its most common denomination TEOS, is one of the most common deposition precursors used in FEBID- and SEM-assisted processes, CVD, ALD, and ALD–CVD for structure deposition at the nanometer scale and in the mask repair industry. With a high oxygen content, the Si(OEt)_4_ is a great candidate to be the chosen precursor for the thin-layer deposition of SiO_2_. Safe, nonexplosive, and nonpoisonous if inhaled in small quantities, the Si(OEt)_4_ is one of the most desired chemical compounds for deposition purposes. Different deposition processes have been used to create thin layers and well-defined structures on surfaces, such as CVD, first used in 1961 for the deposition of TEOS, LPCVD, APCVD, or PECVD,^1^ all differing in the temperature of deposition of the compound and the use of O_2_ or O_3_. During a CVD process of TEOS, the deposition temperature reaches 750 °C; in LPCVD processes, the temperature is reduced to 600 °C, while PECVD with the addition of O_2_ has a nominal temperature of 200 °C releasing and removing CO and CO_2_ and obtaining structures with a higher resistivity of ∼10^16^ Ω·cm. APCVD compared to PECVD or LPCVD has the advantage of the addition of O_3_ to the general deposition process and a high reduction in temperature close to ∼300 °C; the O_3_ traps the TEOS molecules on the surface depositing higher efficiency thin films and structures with lower contamination and lower stress levels.^1^ When using alkoxysilanes as precursors for ALD deposition, the addition of water and the chemisorption of SiH_4_ on a hydrogenated oxide surface is necessary to break the Si–OR bonds by reaction with a hydroxy–OH group, but by using a NH_2_ compound, H_2_N(CH_2_)_3_Si(OEt)_3_,^2^ the deposition of SiO_2_ can take place without the use of a catalyst. Other sources of SiO_2_ used in the deposition of silica and for analysis studies are SiH_3_,^3,4^ SiH_4_,^5^ diethylsilane (Et_2_SiH_2_),^6^ 1,4-dislabutane (DBS, H_3_SiCH_2_CH_2_SiH_3_),^6^ 2,4,6,8-tetramethylcyclotetrasiloxane (TMCTS–R = CH_3_),^6^ and 2,4,6,8-tetraethylcyclotetrasiloxane (TMCTS–R=C_2_H_5_).^6^ In the focused electron beam-induced deposition (FEBID)^7,8^ process with CVD, ALD, and PECVD, the necessity of adding O_2_, O_3_, or any of the hydroxy–OH groups is removed, and the use of the electron beam for the fragmentation and breaking of the bonds to form high-purity deposits of SiO_2_ is proven to be a method with high efficiency and the purity content of the deposits reaching 90 atom %.

### Beam Currents and Deposition Rates

Line profiles and dot profiles (pillars) were done for the first deposition set of Si(OEt)_4_ 8 months old structures. The line profiles heights/widths are presented in Table S1 in the Supporting Information Section. At a first look, the line profiles are better preserved than the dot profiles (Figure 3). The AFM measurements and processed images of the deposits show a merging of the dot profiles and an evolution of their composition to a higher carbon content. The tilting/collapse created in a first instance by the drying of the product and in a second step is the effect of the accumulated moisture from air in conjunction with a much smaller base width compared to the height value. Similar behavior was observed in past experimental work by Randolph et al. in ref (8). The values reported by Randolph and co-workers^8^ are for structures with similar dimensions, deposited at the electron beam energy of 1 keV.

**Figure 4:**
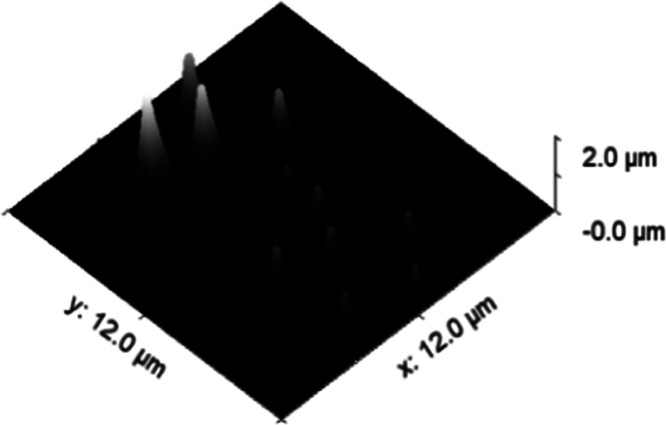
3D view of Si(OEt)_4_ 12 h old deposits.

The orientation of the Au(111) grains of the substrate influences the shape and orientation of the resulting structures on the deposition surface,^9^ asperities, and defects of the surface being often the reason of tilting and collapse of structures, where the deposition rate has a considerable influence on the induced damage on the structure. The width of the deposits is directly proportional to the density of backscattered and secondary electrons emitted from the 1 keV electron beam used for deposition. Accordingly, the halo around the structure and the Si found in the background spectrum would give an indication of how much is deposited near the structure during the structure growth. Values of 0.63 nm average have been found for the Au(111) substrate, while the halo around the structures has a thickness value of 0.015 nm. Two smaller structures (Figure 4) around the main structures have been observed and identified as substrate grains covered by thin layers of precursors.

The 12 h old deposits are characterized by no morphologic modifications due to the accumulation of N_2_ and H_2_O on the surface and to less exposure to air and atmospheric pressure, as well as a smoother surface characterized by less defects and kinks where atoms and parts of the fragments agglomerate.

**Figure 5:**
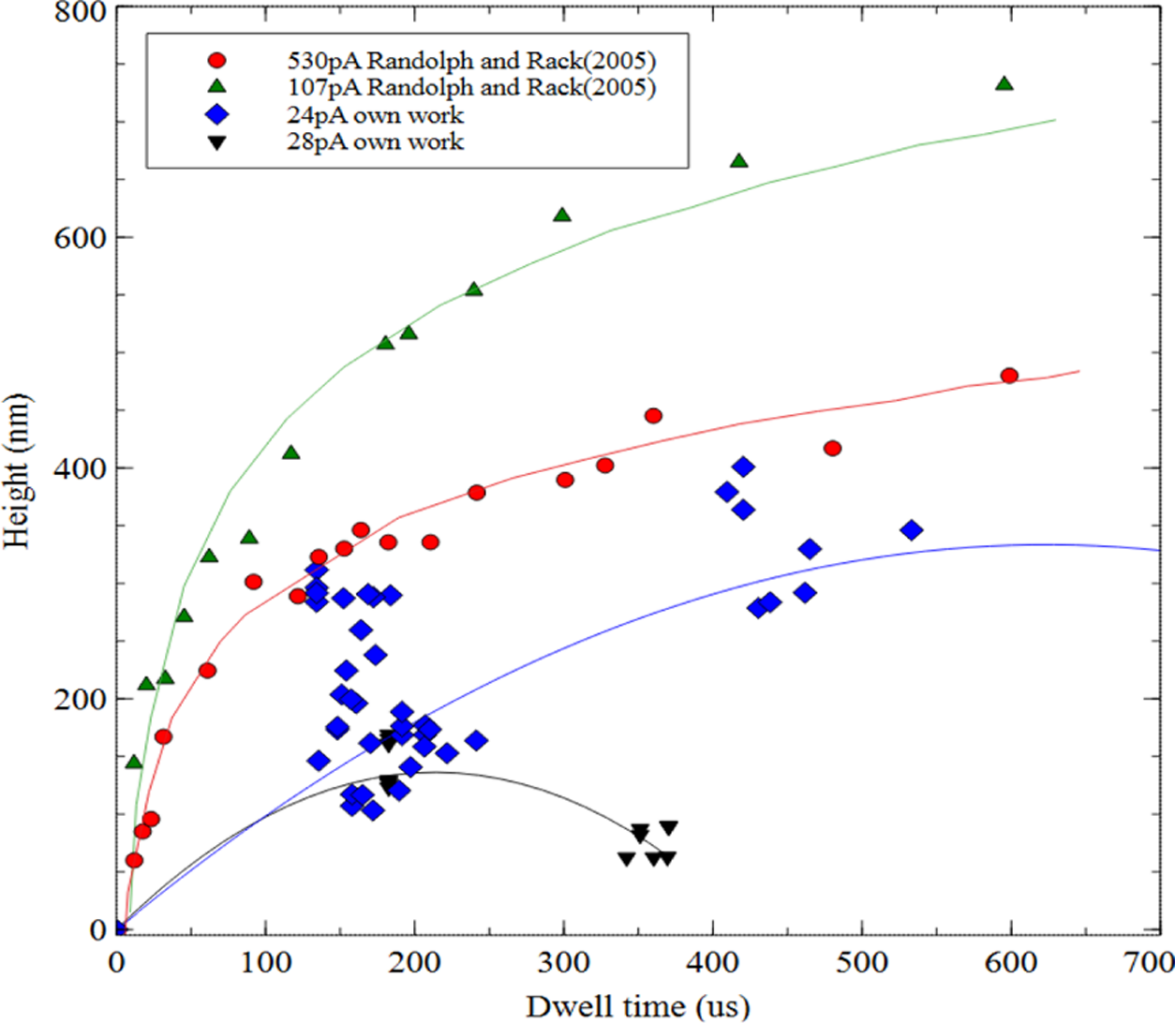
Deposition rates of Si(OEt)_4_ for different beam current values.

The dimensions of the 12 h old deposits are 62.4–89.9/310–341 nm height and 123.1–168.8/350 nm width for a dwell time (μs) of 700 μs/350 μs for the line profiles and 487–1628 nm height and 248–783 nm width for a variable dwell time (μs) of the dot profiles in the range of 250–5030 μs, for a beam characteristic of 1 keV. The 12 h old deposition and background analysis shows a 10 nm substrate to the structure growth compared to the older substrate from the 1st deposition with a value over 630 nm, though around the structures in the 2nd deposition no extra material can be found. Randolph, Fowlkes, and Rack (2005)^8^ declared higher deposition cross-sectional values than obtained from our experimental work, almost double in height for currents of 107 and 530 pA (Figure 5). The deposition times (μs) of the structures and their widths (nm) and heights (nm) are presented in the additional file supporting the article.

### Casino Simulations of Beam Characteristics

**Figure 6:**
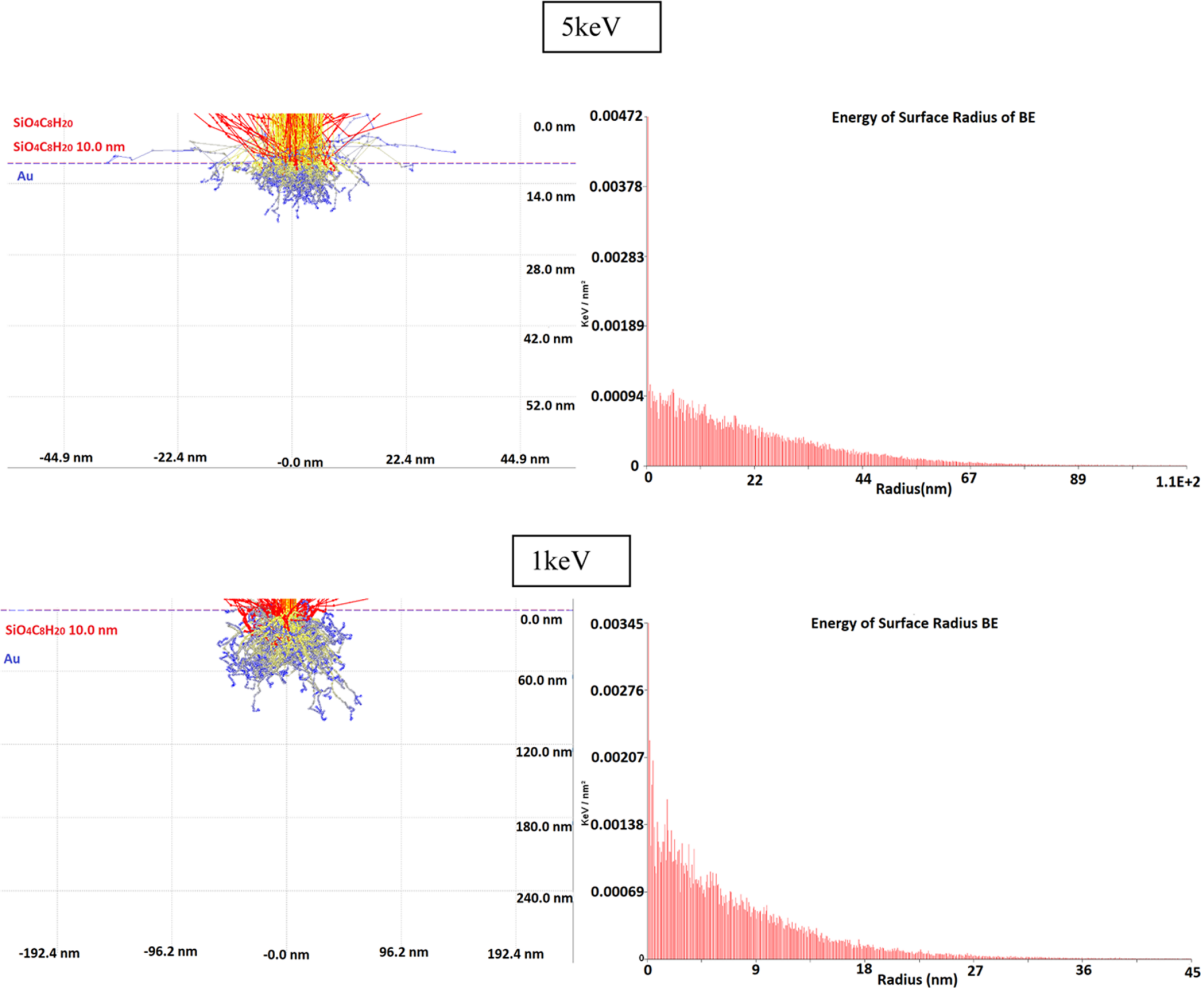
Backscattered electron distribution on the surface at 1 and 5 keV. Electron trajectory simulations at 5 and 1 keV.

Further surface studies involving CASINO simulations (Figure 6) of electron distributions show a maximum radius of visible electrons around the structure of up to 9 nm with the highest distribution between 1 and 2 nm. Backscattered electrons and secondary electrons with energies as high as 200 eV, by breaking ligands and forming additional negative and positive anions, deposit secondary structures around the main structures creating contamination on the substrate, e.g., layers of ethyl and methyl. The simulations at 1 keV have lower backscattered radius than the simulations done at 2 keV. At 5 keV, a backscattered radius of 26 nm is observed, lower in the number of electrons that can create structures over 1 nm^2^.

The CASINO simulations have been run on a predefined Au(111) substrate with the 100 nm × 100 nm × 20 nm and a pyramid set (with Si(OEt)_4_ composition) to intercept the box with 50 nm × 50 nm × 50 nm at angles (70, 90, 70, 90). The distribution of the backscattered electrons and secondary electrons is obtained from the backscattered radius, presenting the length of the density of backscattered electrons around the predefined (0, 0, 0) point of the main beam. The CASINO version used was CASINO v.3.3, with comparison to the CASINO v2.42 for planar area surface distribution (cross section of adsorbed energy of backscattered SEs).

Based on the Monte Carlo routine of electron trajectory calculations, the simulation presents a number of backscattered radii, secondary electron radii, and maximum scattered radii and energy of the backscattered electrons and secondary electrons. Earlier studies of backscattered and secondary electron processes in Mott insulators and cathodoluminescence have been run using CASINO v2.42 simulations,^34,35^ while newer studies focus on the sensitivity of measurement (2020)^36^ and 3D applications in CMOS nanotechnology (2011)^37^ using CASINO v3.3. The software focuses its algorithm on Markov chains^46^ and Voronoi triangulation^47^ and uses the splitting of the nanostructure + substrate in the 3D triangle (meshing) model developed by Akenine–Möller in 1997 and improved by the addition of all of the triangles into a 3D partition tree by Mark de Berg in 2008.^37^ All of the structures have defined an inner shape and an outer shape making possible the declaration of different compositions at the top thin layer (oxidation, substrate–nanostructure interactions, thin-layer effects).

We observed different distributions of the BSEs on the structure/Au(111) with the change in the PEs energy from 2 to 5 keV (see Table 3) almost 50% in both cases, while for the 1 keV, the radius is 10 times lower, though the energy of the BSEs on the surface is higher than for the 2 keV simulation. Lower energy is observed in the case of the 5 keV simulations of 0.134 keV/nm^2^ close in value to the energy obtained for the 1 keV beam of 1.121 keV/nm^2^. Simulations of higher energies up to 10 keV have been done with CASINO v2.42 in thin Si(OEt)_4_ layers of no more than 10 nm with the energy of the surface radius of 0.00253 keV/nm^2^ and BSEs falling between 8 and 10 keV.

**Table 3:** Results of Electron Trajectory Simulations Using CASINO Software for 1, 2, and 5 keV

beam voltage (keV)	maximum energy of surface radius (hits/nm^2^)	backscattered radius (nm)	energy of surface radius of BSE (keV/nm^2^)
1	0.151	1.2	0.121
2	0.05316	12.2	0.08172
5	0.03617	26	0.134

To obtain the energy distribution of backscattered electrons, the sample was declared as a 10 nm layer with SiO_4_C_8_H_20_ composition deposited on an Au(111) substrate using CASINO v2.42, for a number of 200 displayed trajectories. For the total and partial cross sections of the electron distributions, the two models of Drouin and Gauvin (1993) and the ionization potentials from the Joy and Luo model (1989) have been used.

**Figure 7:**
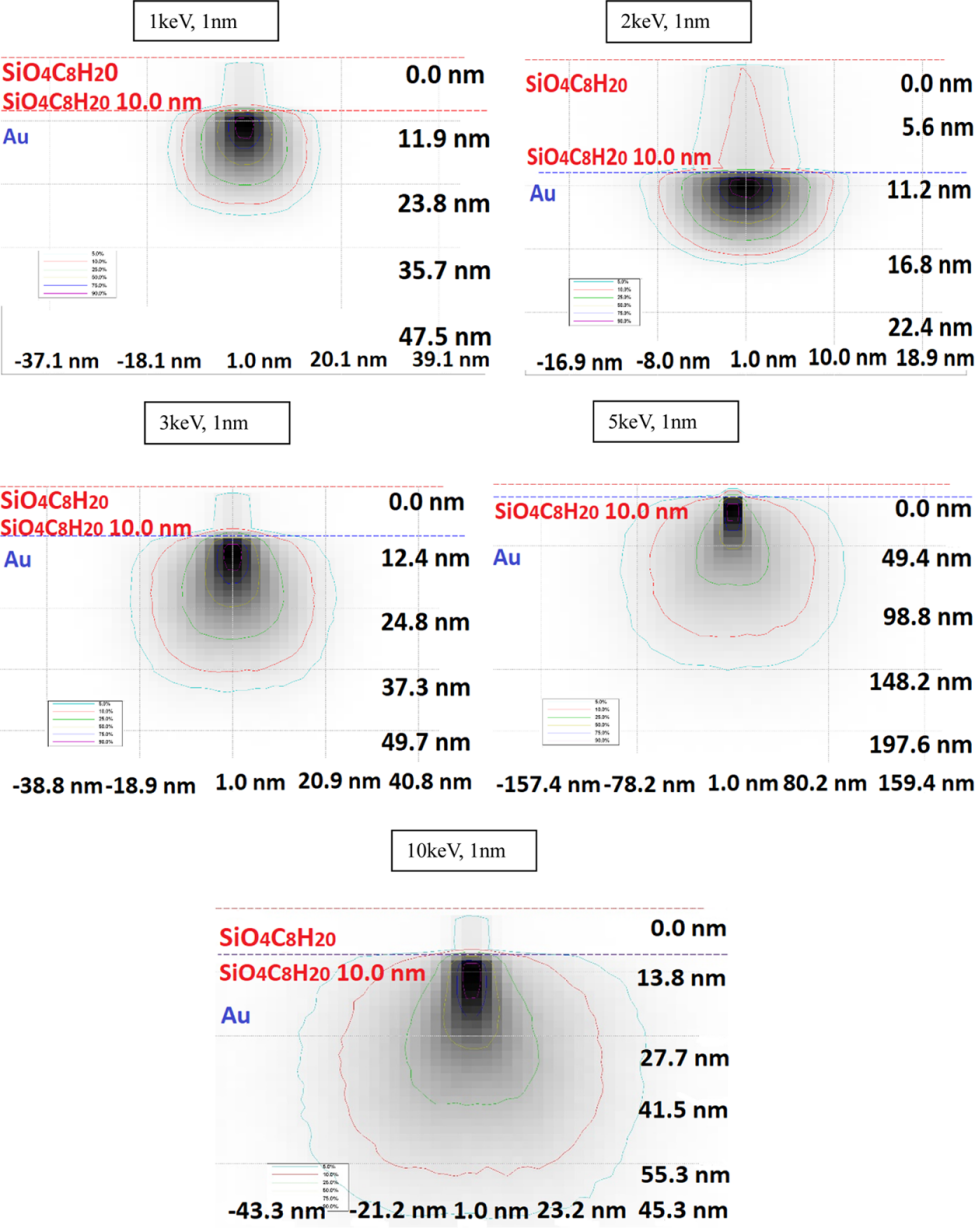
Simulated absorbed energy of the sample and substrate at 1–10 keV and 1 nm from the center of the sample.

The simulated absorbed energy is presented in Figure 7; it can easily be observed that for 1, 3, and 5 keV, the presence of 10% distribution lines is lower in the nanostructures compared to 2 and 10 keV where the 10 and 5% lines go upper in the nanostructures with sharper peaks. For 1 and 10 keV, we observe contributions from the 25% lines.

### Deposit Analysis

**Figure 8:**
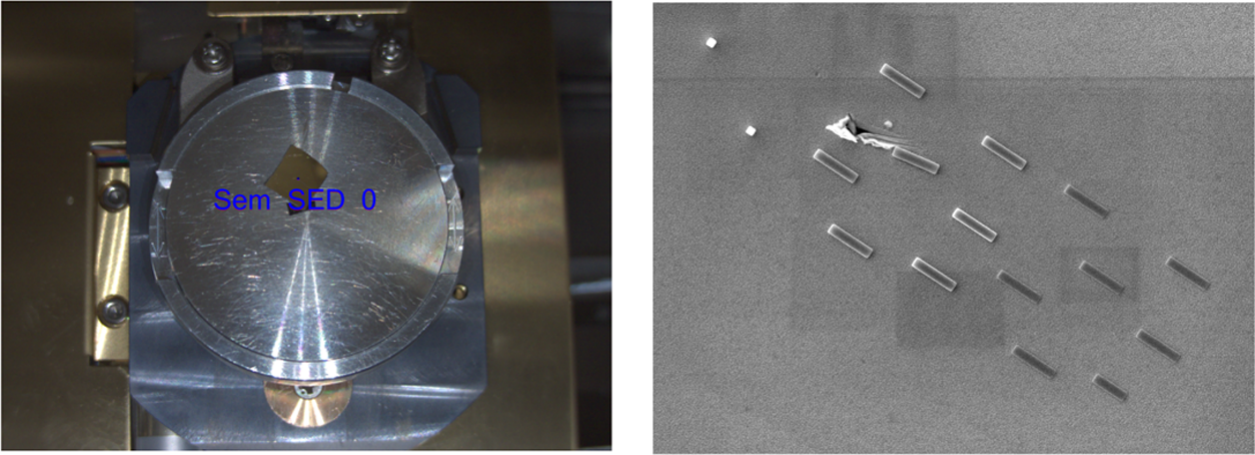
Si(OEt)_4_ on Au(111)/SiO_2_ 12 months old sample (left) and top view of the deposits (right).

**Figure 9:**
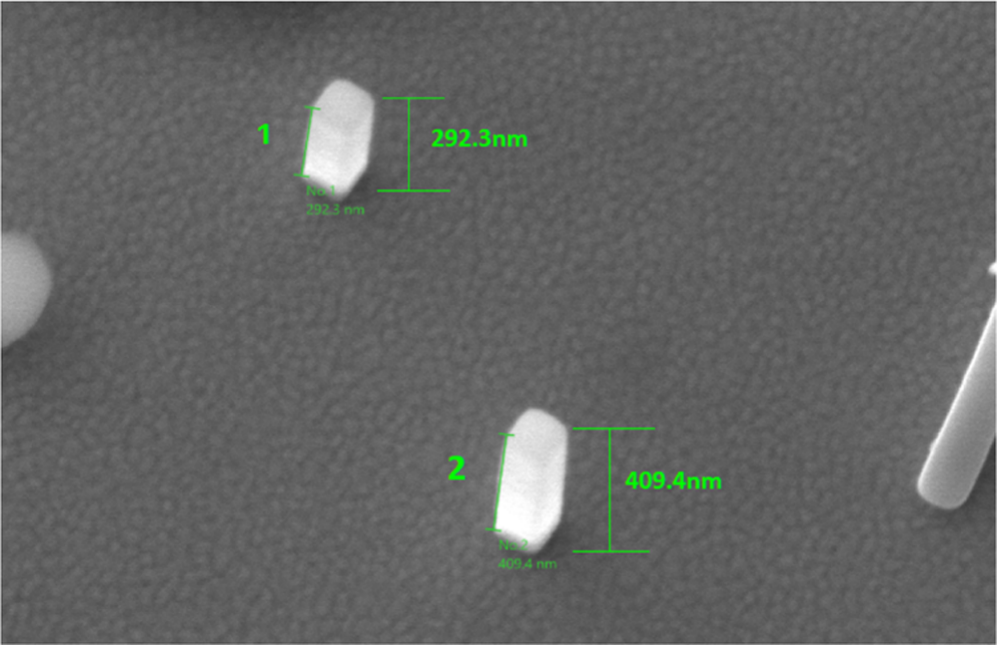
Tilted view to 15° of the Si(OEt)_4_ pillar shape structures of 12 months old deposits.

A second set of measurements was carried on the 12 h old deposits after a period of 12 months (Figure 8). The deposits suffered modification over time due to storage and handling, as well as due to the aging of the substrate. The packing of the Au atoms modified over time, creating holes and kinks in the structures and undergoing in particular area phenomena as tasation (settlement), integration, and reorientation.^42,43^ We do not assign the defects on the substrate to the transport or handling but to the modifications and reorganization of the Au atoms under an ambient atmosphere and room temperature. Some of the structures collapsed as a result of prolonged exposure to air. The deposits have been measured at 10 and 15° stage tilt to obtain the compositional content of the structures. The heights of the structures were obtained with a certain degree of error, as the EDX measurement is not intended for verification of the nanodeposit height (Figure 9).

A separate AFM study to determine the height of the structures was run, ranging in values from 70 nm (the smallest) in the shape of a line to 1.684 μm (the largest) in the shape of a pillar and a very large base, almost equal in dimension with the height, with a value of ∼1.2 μm.

**Figure 10:**
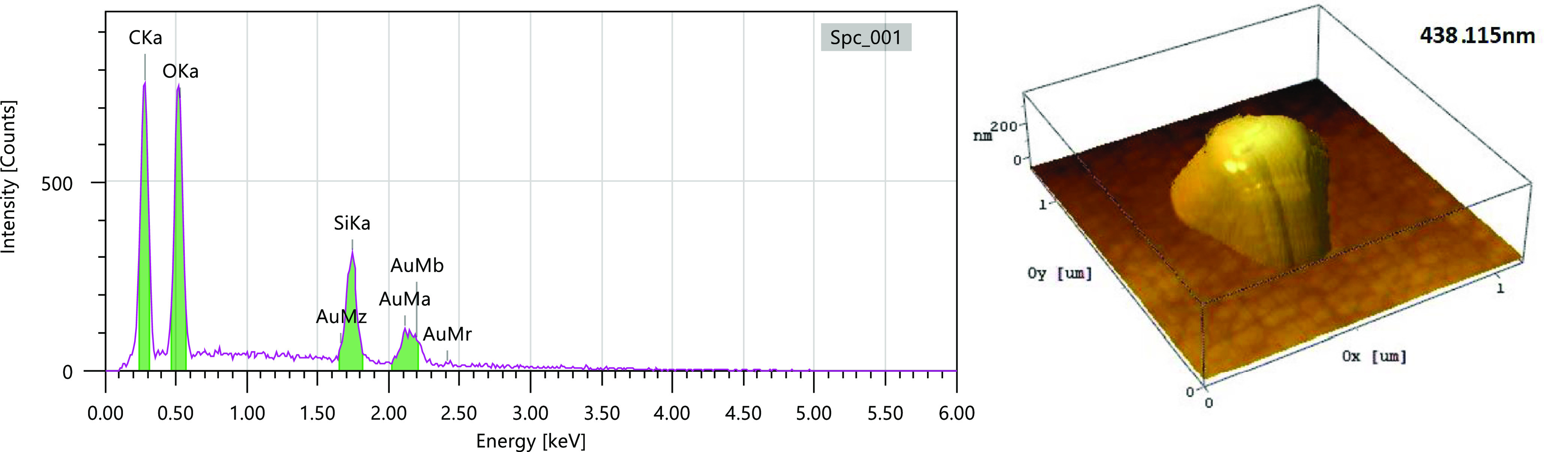
Compositional analysis of structure 2 from Figure 5 using EDX with the AFM view of height.

For the structure 2 in Figure 9, an EDX compositional analysis was run (see Figure 10) with resulting C content up to 21, 26.11 wt % O content, and 17.91 wt % Si content, with the atomic ratios of 41.73 atom % C, 38.86 atom % O, and 15.19 atom % Si. The presence of Au is due to the thin layer of deposit, EDX acquiring data up to 100 nm in the substrate.

**Figure 11:**
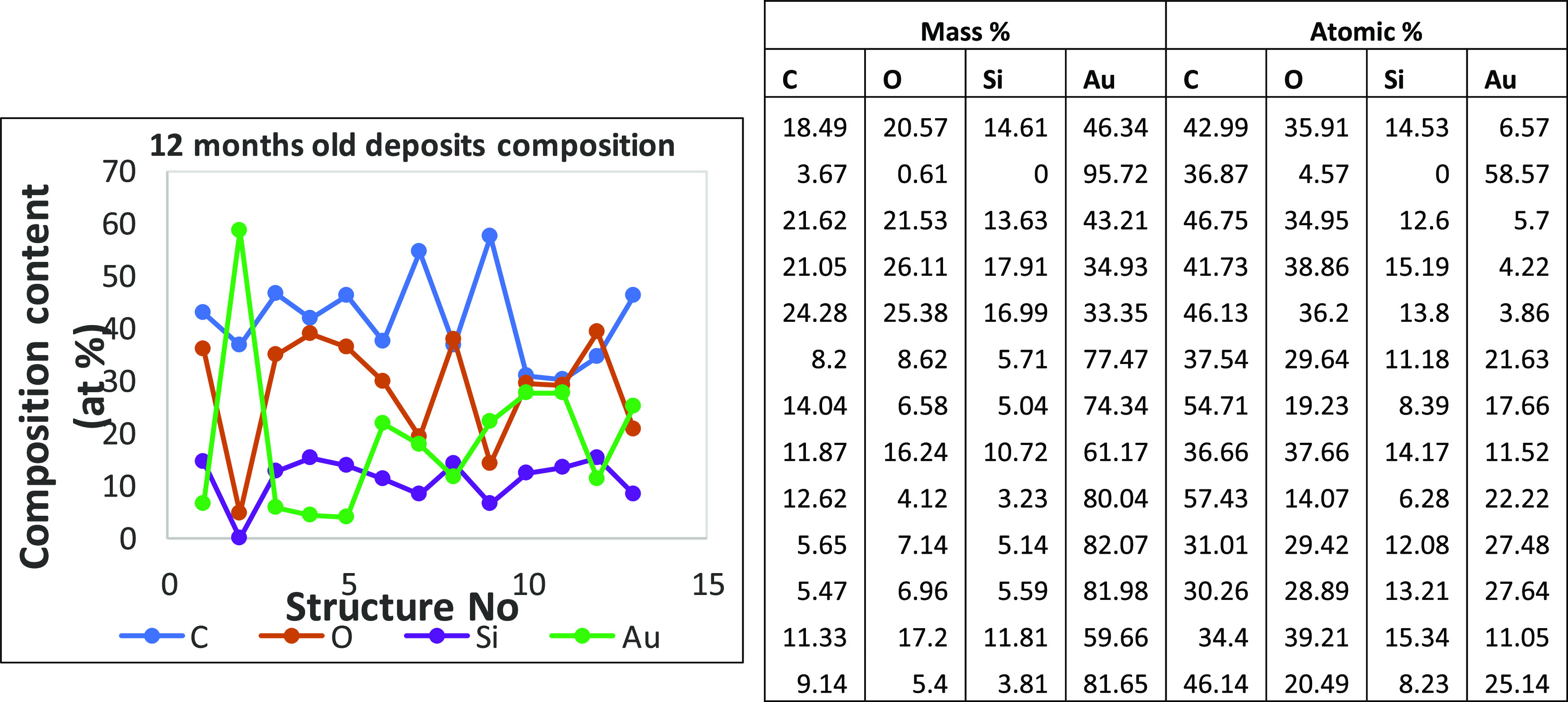
Atomic % elemental content of 12 months old structures.

The intent is to build structures with high SiO_2_ purity, though limited by the high C content and the unavailability to use H_2_, O_2_, or H_2_O jets to purify the structure.^37,39^ Geier et al.^39^ report the full removal of C content of deposited Pt structures using H_2_O vapor jets. As transition element and not a metal, the rate of C desorption from the substrate is reduced with the H_2_O vapor/O_2_ jets compared to metals but still presenting a significant reduction and sensitivity to the process.^40^ An increase in the Si and the height of the structure is observed with dwell time, but limited by the high C content of the nanostructures; in comparison, line profiles have higher C content due to their horizontal growth compared to the vertical growth of the pillar profiles. The C contents reported from our experimental measurements on our lines and pillar profiles have minimum values of 40 atom % C, with the highest value recorded of 57.43 atom % C. The second structure (Figure 10) has 0 atom % Si composition that we assign to the missing of the structure and deformation of the surface, the EDX being able to identify in this case only contamination gas from air. An average value of 12.08 atom % composition of Si is obtained from the 12 structures (Figure 11); the one structure without any Si was not considered for the average calculation.

**Figure 12:**
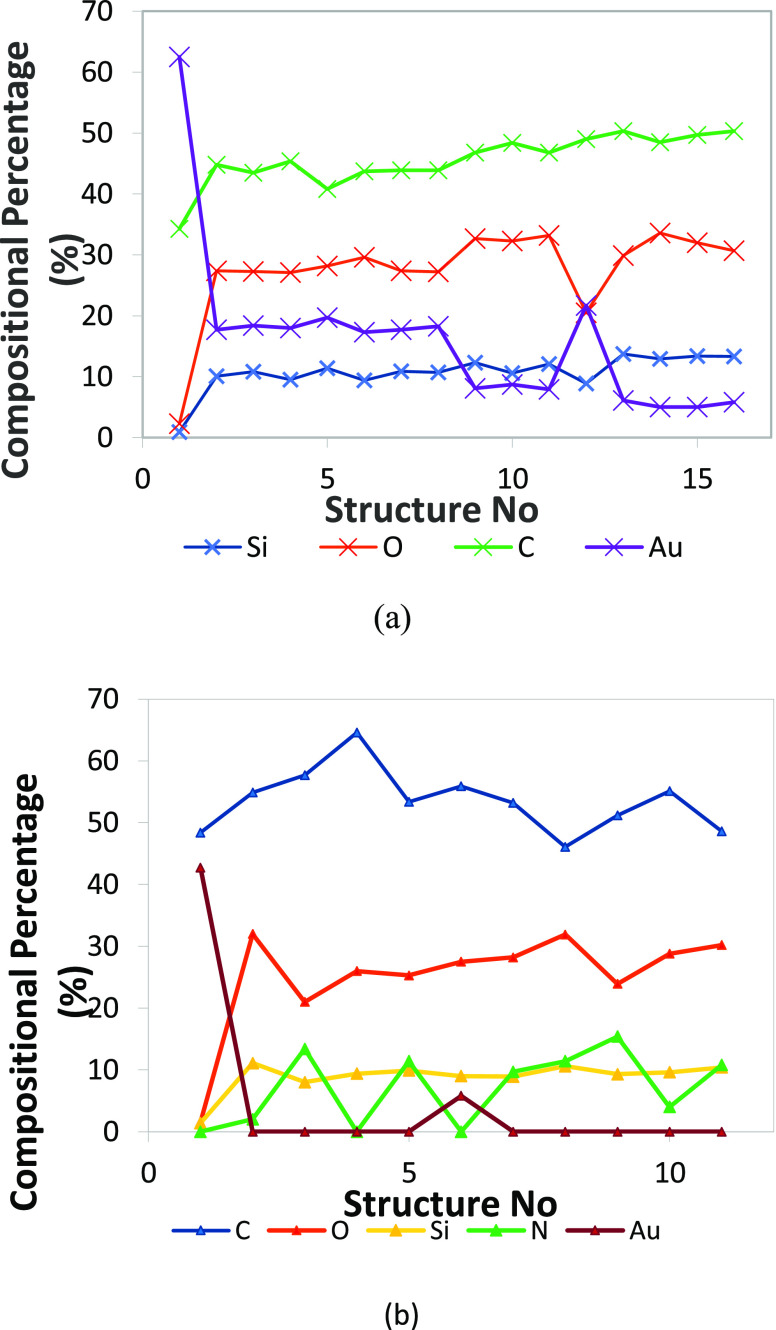
Elemental composition of the Si(OEt)_4_-deposited nanostructures: 12 h old deposits (a) and 8 months old deposits on irregular Au surface (b).

The EDX composition analysis reveals the presence of four elements in all structures, Si, O, C, and extra N, due to contamination and prolonged exposure to air. In Figure 12, the 8 months old deposit compositions and 12 h old deposit compositions are presented. A higher C concentration is observed, with a presence of Si of only 12 atom % without the addition of other gases during the deposition process. The content of Si increases for the 12 h old deposits up to values of 16–17 atom %, but still lower than the reported values of 32 atom % for a pure SiO_2_ structure.

Structures (in Figure 12) of 8 months old deposits have the Au content with values less than 5 atom %, while the 12 h old deposits have a higher level of Au signal contribution in the compositional analysis of the deposits. The difference between the two measurements suggests the presence of two compositional different Au(111)/SiO_2_ surfaces, a 200 nm thick Au(111) layer on the SiO_2_ 8 months old substrate, and a 100 nm Au(111) layer on SiO_2_ for the 12 h old structures and substrate. The changes in the composition and dimensions of the structures that would be the sign of oxidation and reactivity with the presence of moisture on the substrate from air are not observed and are limited in magnitude, without a great impact on the structures over time. Higher C contents are observed for the older structures, which cannot come from the C contained in the Si(OEt)_4_ at the deposition time, but to the accumulated C during the 8 months exposure to air.

**Figure 13:**
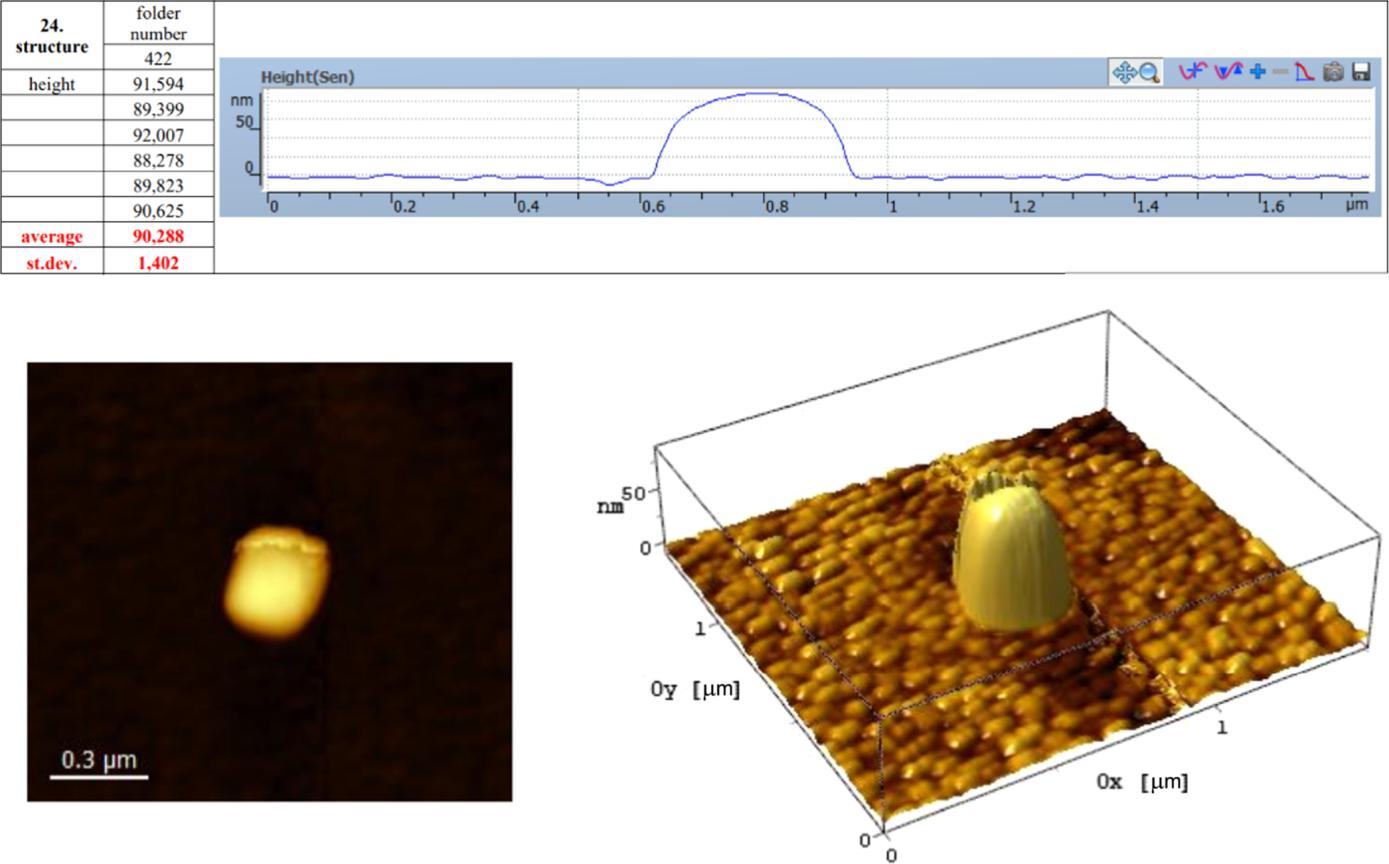
AFM measurement of pillar structure 1 in Figure 10. The magnitude of the structural modifications in the pillar can be observed through changes in the height and composition of the nanostructures.

For the present structure (Figure 13), no oxidation is observed that would create changes in the morphology, shape, and radical changes in height. The height of the structure is 92.007 nm at the highest point with an average height of 90.288 nm. A substrate with high granulation starts to appear as a result of aging of the SiO_2_/Au(111) surface; it is known for the Au(111) substrates to present high Au granulation organized in Chevron patterns on the surface, a behavior presented in detail in the works of Allmers and Donath^41^ who developed a study of the substrate reconstruction of the Au(111) surface. A lateral width of the deposit of 0.32 μm is observed; the growth of the pillar was higher in plane than vertically.

For the 12 h old structures, the C content is under 50 atom %, while the C content in the case of the 8 months old deposits increases to over 60 atom %. Another sign of exposure to air of the deposited structure is the presence of small amounts of other atmospheric gases regularly found in the breathable air in rooms, as N_2_. The H_2_O molecules cannot be determined as water singlets, dimers, or trimers using EDX analysis, but an increase in the O content is obtained. The structures on a new, clean, and undamaged Au(111) substrate are unlikely to be affected by H_2_O in air. The Si–O bonds are strong bonds with dissociation energies in the range of ∼452 kJ/mol, while a H–O bond has a value of 467 kJ/mol; it is likely for the Si–O bonds to break and form bonds to O atoms from H_2_O and to integrate H_2_O molecules in the structures.

**Figure 14:**
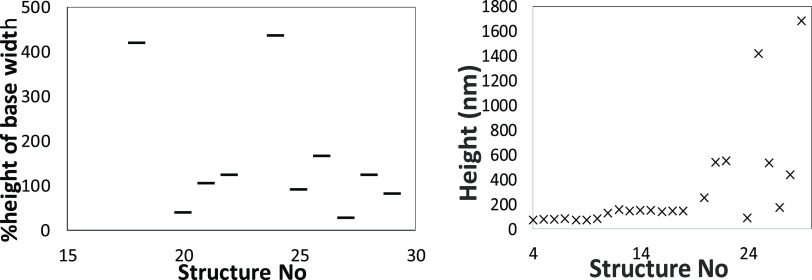
Height of 12 months old structures with the increase of dwell time from 350 to 5030 μs (right) and % height of the base width (left). The width of the structures (see % height of the base width) is linearly proportional with the height of the structures. A good example is structure 29 that has the width of the base close to the value of the height of the structure.

The strength of the SiO_2_ reduces with the water content and moisture in the atmosphere and is a softer material^19,20^ compared to the regular metal nanometer structures, rendering it hard to be grown on the vertical. The extent of the pillar base increases with the increase in the height of the structure, while Au, Pt, Fe, and Co compounds hardly increase the base when vertically deposited. With heights between 60 nm and 1.7 μm, the 12 months old structures have conserved up to 80% intact; two of the structures collapsed during the 12 months, assigned to a lower width of the base. Figure 14 presents a comparison of the heights with the base widths of the structure, a thinner base percentage compared with the height creating an unstable structure that due to the softness of the material^21,22^ leads to the collapse of the pillar structures. The line profiles have preserved intact up to 100% of all deposited structures. The substrate had no influence on the growth of the nanostructures as it was newly purchased, in very good condition without visible grains and tasation. A different effect (height reduction, structure contamination) is observed for the 8 months old deposits.

In Table 4, structures 20 and 27 are the two structures that tilted reducing the conservation percentage of all of the nanostructures to 80%. There is a clear difference in the % height of base width compared to the other structures all having over 60% values. Structure 20 presents a value of 36.52% of the base width value compared to the height of the structure, while structure 27 presents a value of 23.72% of the base width value compared to the height of the structure. Percentages lower than 60% increase the possibility of tilting and collapse of the pillar nanostructures, indicating that these structures were created with low dwell time (us), high number of deposition loops (2600), and short standby time (see Table 2 for values). A higher dwell time with the same number of loops increases the % height of base width to over 120%, while lowering the loops (1000) and increasing the dwell time (μs) would create very wide bases of the nanopillars with values of the % height of base width of over ∼400%.

**Table 4:** 12 Months Old Deposits % Height of the Base Width of Pillar Structures

structure (no)	max height (nm)	base width (nm)	% height of base width
18	155.176	650	418.88
20	840	306.749	36.52
21	544.926	560	102.77
22	594.941	720	121.02
24	92.007	400	434.75
25	1439.5	1260	87.53
26	583.737	950	162.74
27	1390	329.679	23.72
28	438.115	530	120.97
29	1720.31	1360	79.06

**Figure 15:**
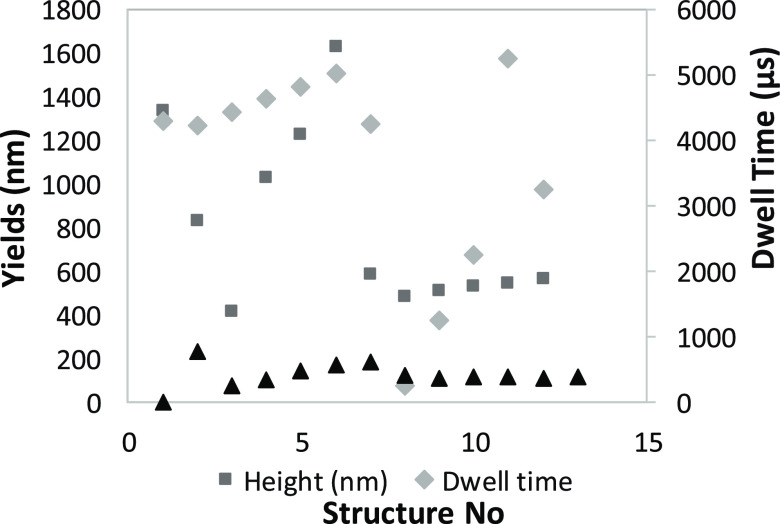
Height/width with a dwell time of 8 months old nanopillar structures (peak values are presented in the Supporting Information).

**Figure 16:**
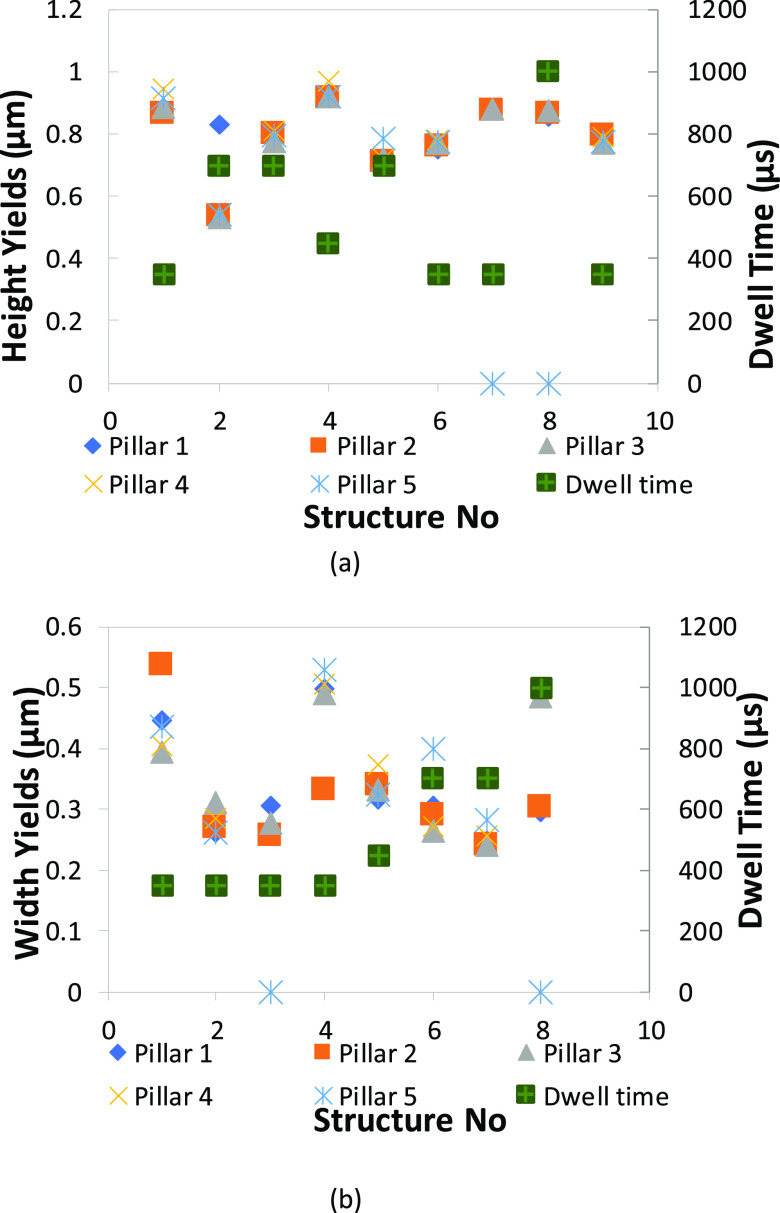
Height/width with a dwell time of 12 h old structures: (a) height (μm) and (b) width (μm).

The decrease in height with the increase in the dwell time observed at 750 and 1200 μs (Figure 15) compared to the height regime observed at 350 and 500 μs where with the increase in time, an increase in the structure height is observed corresponding to a beam-limited growth regime.^8,10−18^ For an average 1 keV beam current, the dwell time limit to suppress the growth is set to around 700 μs. With applications in biomedical and biosensing applications, the SiO_2_ frameworks enhanced with organic materials for mineralization of bone tissues and DNA^46^ deposited at the nanoscale, and their growth and mineralization make the focus of highly increasing in importance studies on inhomogeneous (e.g., Au(111) substrate with defects) and homogeneous (perfect hcp lattice) substrate depositions. Defects in the Au(111) surface are filled with the SiO_2_C_x_ material through C bonds between the C in the defects and the remaining C of the deposited molecules. Rolling of already formed SiO_2_ can take place with higher energy consumption through vaporization and electron excitation of the formed ions. The bonding with O-layers at the surface is highly improbable^48^ as the Au(111) surface would need to undergo an oxidation process, while the most probable process is the integration of Si atoms in the substrate at the deposition moment (Figure 16).

The same behavior as seen in Figure 15 is observed for the 12 h old deposits with even more pronounced differences. The behavior does not change with the analysis of the width increase and decrease; the lateral growth follows the same path, and it is limited by the dwell time and the beam current magnitude. With a Si/O ratio content of 11:28, the 12 months old structure exhibits slightly less height and width, still having a high amount of C content, in a process of deposition that did not use additives for purification of deposits. No further purification step was done for the nanostructures using either O_2_ or H_2_O jets. A common behavior is the increase in the C content of the structures following exposure to air and room temperature. Sánchez et al.^47^ analyzed the process of deposition using electron beam and ion beam depositions with higher Si/O (15:33) ratios with the addition of H_2_O in the deposition process. Plank et al.^45^ comparing their deposition studies to the ones of Sánchez et al.^47^ declared structures of less than 10 nm deposited in thin layers (used for coatings in the manufacturing industry) and with composition close to the composition of pure SiO_2_. A higher Si/O ratio (Figure 12) is observed in good agreement with refs (45) and (47) for the 12 h old structures with an average of 11:29.

**Figure 17:**
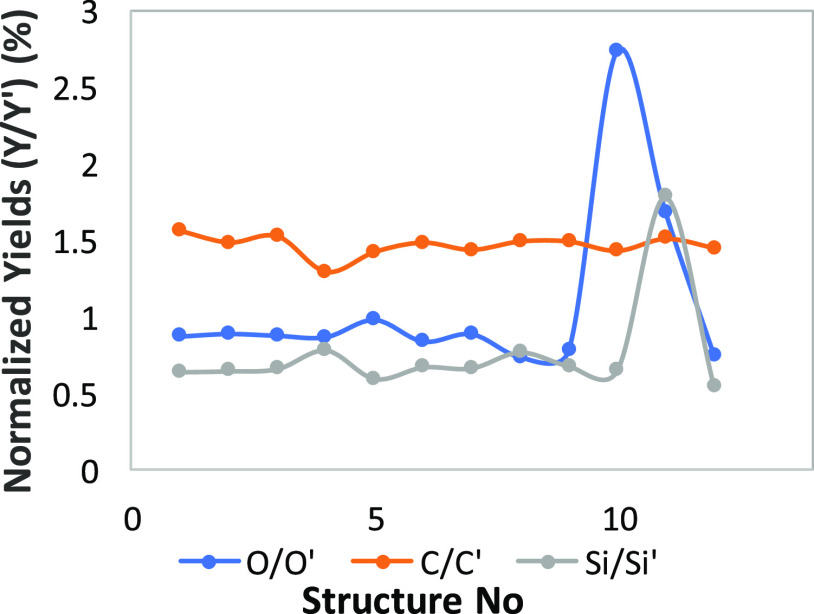
Ratios of C, O, and Si for 12 months old deposits (′) and 12 h old deposits ().

For values under 1, the ratios of the 12 months old deposits compared with the 12 h old deposits show an increase in the compositional content of the structure with the measured element. The C/C′ (Figure 17) presents the expected behavior; with time, the carbon content of the structures increases, but the O and Si present a lower increase at the atom % composition of the structures, caused by a phenomenon of evaporation or inoculation of O and Si in the presence of H_2_O and N_2_ from air.

## Conclusions

The study wants to be a complex study of the composition and structure of the Si(OEt)_4_ nanostructures on Au(111)/SiO_2_ substrates, looking at the behavior of the compound on the substrate, more than an intent to reach the smallest nanometer size structure. A moisture ratio to the base–height of the pillar was not obtained as a result of the study. While the deposition temperature would help control better the shape of the structures, important parameters such as the structural parameters (height with base, for pillar shape) combined with beam characteristics would give optimum deposition. The present study wants to bring up and discuss the different conditions for the deposition of Si(OEt)_4_, from the deposition temperature to time and exposure to the electron beams and air.

The behavior of the Si(OEt)_4_ at FEBID deposition was repeatedly studied by scientist in different conditions as the precursor is one of the widest used precursors for the deposition of Si and SiO_2_. The accumulation of moisture through the high number of O atoms in the deposits coming from H_2_O creates problems at the deposition of pillar shape structures especially when the base of the structure is much smaller than the height of the structure.

Results have been obtained for structures between 9 nm and up to 0.5 μm with success, though the deposition of very small Si(OEt)_4_ structures, less than 6 nm, still remains a quest. Higher C and O, with the presence of N from N_2_, were obtained at the comparison of the depositions of 12 h and 12 months old deposits. The aging of the substrate brings substantial changes to the structures with an increase in the C content up to 10%. Even though it is a widely used compound for deposition, high carbon contents of 50 atom % have been obtained for the 12 h old nanostructures and 66 atom % for 8 months old nanostructures, suggesting that a second annealing step would be needed. Further studies of the method with the addition of O_2_/H_2_O jets could show an improvement in the purity of SiO_2_ nanodeposits.

## Supporting Information Available

Widths and heights of 8 months old nanostructures (PDF)
