# Supplementary material for: Structural Analysis of Si(OEt)4 Deposits on Au(111)/SiO2 Substrates at the Nanometer Scale Using Focused Electron Beam-Induced Deposition
Source: ACS Omega. 2023 Jun 28;8(27):24233–46. doi: 10.1021/acsomega.3c00793 (PMC10339401; doi:10.1021/acsomega.3c00793)
Supplement: Supplementary file 1 — ao3c00793_si_001.pdf [file ao3c00793_si_001.pdf]

Structural Analysis of $Si(OEt)_4$ Deposits on Au(111)/SiO_2 Substrates at Nanometer Scale using Focused Electron Beam Induced Deposition

[1]Nigel J. Mason, [1]Maria Pintea*, [2]István Csarnovics, [3]Tamás Fodor, [3]Zita Szikszai, [3]Zsófia Kertész

[1]University of Kent, School of Physical Sciences, Canterbury, United Kingdom

[2]Debreceni Egyetem Termeszettudomanyi es Technologiai Kar, Department of Experimental Physics

[3]Laboratory of Materials Science, Institute for Nuclear Research, Debrecen, Hungary

Supporting Information: Widths and heights of 8 months old nanostructures.

Type of deposit	Dwell Time (us)	Loops (number)	Beam current [pA]
6 points	1.325/ 2.456/ 3.564/ 4.634/ 5.741/ 6.880	345/ 1102/ 2203/ 3301/ 4112/ 5734	24
6 points	10.325/ 12.456/ 13.564/ 14.634/ 15.741/ 16.886	1345/ 2102/ 3203/ 4301/ 5112/ 6734	24
6 points	20.325/ 22.456/ 23.564/ 24.634/ 25.741/ 26.886	10345/ 12102/ 13203/ 14301/ 15112/ 16734 & 1000	24
6 points	30.325/ 32.456/ 33.564/ 34.634/ 35.741/ 36.886	1000	24
6 points	40.325/ 42.456/ 43.564/ 44.634/ 45.741/ 46.886	1000	24
6 points	50.325/ 52.456/ 53.564/ 54.634/ 55.741/ 56.886	1000	24
6 points	80.325/ 82.450/ 83.564/ 84.634/ 85.741/ 86.886	1000	24
6 points	2.350/ 12.350/ 22.350/ 32.350/ 42.350/ 52.350	1000, 1300, 2000, 2500, 3000, 1800, 1500, 4000	24
6 points	1/ 5/ 10/ 15/ 20/ 25	4000	24
6 points	30/ 35/ 40/ 45/ 50/ 55	4000	24
6 points	20.325/ 22.325/ 24.325/ 26.325/ 28.325/ 30.325	1300	24
6 points	10.325/ 12.325/ 14.325/ 16.325/ 18.325/ 20.325	1300	24
6 points	30.325/ 32.325/ 34.325/ 36.325/ 38.325/ 40.325	1300	24
6 points	16	1300	24
6 points	30	1300	24
6 points	10	1300	24
6 points	20	1300	24
6 lines	5	1300	24
6 lines	0.7	500	24
6 lines	0.45	500	24

6 lines	0.7	700	24
6 lines	0.35	1300	24

Table S1. 8 months old structures height/width of profiles
